# Linking perceived social support to self-esteem and social integration among adolescents with visual impairment: A cross-lagged study

**DOI:** 10.3389/fpsyg.2022.1054857

**Published:** 2023-01-09

**Authors:** Wei Yuan, Zhengli Xie, Ping Dong, Yuqin Yang

**Affiliations:** ^1^School of Education, Central China Normal University, Wuhan, China; ^2^Faculty of Education, The University of Hong Kong, Pokfulam, Hong Kong SAR, China; ^3^Southampton Education School, University of Southampton, Southampton, United Kingdom; ^4^Faculty of Artificial Intelligence in Education, Central China Normal University, Wuhan, China

**Keywords:** adolescents with visual impairment, perceived social support, self-esteem, social integration, cross-lagged panel modeling

## Abstract

This study examines the relationship between perceived social support and self-esteem and between perceived social support and social integration among adolescents with visual impairments. Adolescents with visual impairments (*N*_time1_ = 311, *N*_time2_ = 170) from four special education schools in eastern China participated in this study within a 1-year interval. The Child and Adolescent Social Support Scale, Rosenberg Self-esteem Questionnaire, and Interpersonal Adaptation Scale were used to collect data. The results from cross-lagged panel modeling showed reciprocal positive relationships between parental support and self-esteem. Self-esteem at T1 positively predicted three other sources of perceived social support at T2: teacher support, classmate support, and close-friend support. Social integration at T1 positively predicted close-friend support at T2. This study extends understanding of the relationships among perceived social support, self-esteem, and social integration, and provides practical implications for parents, schools, and communities to improve psychosocial outcomes in adolescents with visual impairment.

## Introduction

Individuals experience dramatic changes in physiology and psychology during their adolescence and their psychosocial adaptation is susceptible to the influence of the external environment (Deng et al., 2013). Visual impairment has a significant impact on adolescents’ mental development. For example, adolescents with visual impairments tend to have more psychological conflicts (e.g., insecurity about the physical environment, feeling guilt, anxiety, sadness, and depression), and a higher risk of social isolation (e.g., limited involvement in social interaction, barriers in social lives, and loneliness experiences) than sighted adolescents (de Verdier, 2016; Hamurcu et al., 2016; Veerman et al., 2019; Zhang and Xiao, 2020). Therefore, it is necessary to pay attention to the psychosocial adaptation of adolescents with visual impairment.

It has been documented in the literature that social support contributed to socioemotional development of students with visual impairment (see Manitsa and Doikou, 2022 for a detailed review). However, existing studies are mainly cross-sectional, and research on the unique influence of various sources of social support is insufficient. Longitudinal studies are needed to deepen the understanding of relationships between various sources of social support and adolescents’ psychosocial adaptation to visual impairment. Self-esteem and social integration are the two key indicators of psychosocial adaptation in individuals with disabilities (Livneh and Antonak, 1997), and are also the focus of research on adolescents with visual impairment. Therefore, the present study pursues a longitudinal design to address the linkage between perceived social support and self-esteem and that between perceived social support and social integration.

### Perceived social support of adolescents with visual impairment

Thoits (1986, p. 417) defined social support as “functions performed for a distressed individual by significant others such as family members, friends, co-workers, relatives, and neighbors,” indicating that social support has multiple sources. Perceived social support refers to the subjective perceptions and beliefs about the social support received by individuals (Lakey and Scoboria, 2005). Based on their integrative literature review, Manitsa and Doikou (2022) summarized the major themes in existing research on social support for students with visual impairment including their experiences of social support in schools and universities and the impact of these social support on their academic learning and socioemotional development. Research comparing perceived social support reported by adolescents with visual impairment with that reported by sighted adolescents showed inconsistent findings (Pinquart and Pfeiffer, 2013; Hadidi and Al Khateeb, 2014), which may be attributed to social culture, educational placement, and construct conceptualization differences. However, little is known about perceived social support of adolescents with visual impairment in the Chinese context.

Studies outside China found that peer support had greater significant predictive effect on mental health and loneliness of adolescents with visual impairment than parent support (Kef and Deković, 2004; Heppe et al., 2020), which contradicts findings from other studies among sighted adolescents (Rueger et al., 2010). The published work mainly focused on students with visual impairment attending regular schools and largely overlooked those attending special schools. In addition, previous research has rarely discussed the contribution of perceived close-friend support and teacher support among adolescents with visual impairment. It has only been reported that their perceived teacher support is significantly lower than parental and peer support, and that adolescents with visual impairment perceived higher levels of teacher support than their sighted peers (Pinquart and Pfeiffer, 2013). In addition, friends’ social support was found to be a significant predictor of depression of young adults with visual impairment (Papadopoulos and Papakonstantinou, 2020). To better understand the function of social networks among adolescents with visual impairment, it is necessary to further investigate the unique contribution of different sources of social support. Therefore, this study examined the relationships between four sources of perceived social support (i.e., parental, teacher, peer, and close-friend support) and self-esteem, as well as those between the four aforementioned perceived social support and social integration among Chinese adolescents with visual impairments.

### Self-esteem and perceived social support

Self-esteem refers to how much value individuals place on themselves (Rosenberg, 1965). People with higher levels of self-esteem tend to have higher levels of happiness and achieve greater success in their work and social relationships (Orth and Robins, 2022). Self-esteem of children and young adults with visual impairment has attracted research attention. Augestad (2017) did a systematic review on 26 relevant studies between 1998 and 2016 and pointed out that research results were divergent regarding whether the visually impaired would show lower level of self-esteem than the sighted, several factors such as social support, gender, and parenting seemed important for their self-esteem development; however, longitudinal studies are warranted to draw conclusions about causal effects. It is generally acknowledged that adolescents with visual impairment face great challenges to obtain high levels of self-esteem (Miklyaeva and Gorkovaya, 2018), although they may or may not show lower levels of self-esteem than sighted adolescents. A recent study reported that over 90% of students with visual impairment in regular schools in Tanzania had high self-esteem (Kapinga and Aloni, 2021). This result should be carefully interpreted because the sample size was relatively small and the psychometric properties of self-esteem instrument were not reported. Self-esteem of students with visual impairment has also been found to be associated with coping strategies (Yuan et al., 2017; Tołczyk and Pisula, 2019). Xie et al.’s (2022) longitudinal study revealed that lower level of self-esteem among students with visual impairment is a consequence of relinquished control coping and social isolation, and is also a predictor of less adaptive coping strategies over time. The present study continues this line of research by examining the reciprocal relationship between self-esteem and perceived social support among adolescents with visual impairment.

The relationship between perceived social support and self-esteem has gained much attention, and there are three major perspectives concerning its directions. First, social support is the antecedent of self-esteem. According to sociometer theory, the motivation of individuals seeking to increase their relationship value and social acceptance is an important source of self-esteem (Leary, 2005). Dated empirical evidence from Europe suggests that perceived social support significantly predicts self-esteem among adolescents with visual impairment (Huurre et al., 1999; Kef, 2002). It is necessary to revisit this topic in different social–cultural contexts.

Second, self-esteem is the antecedent of perceived social support. According to Brockner’s (1988) plasticity hypothesis, people with low self-esteem are often more sensitive to others’ evaluation and social feedback, thus affecting their social relations. This notion is supported by studies concerning perceived social support’s mediating role in the relationships between self-esteem and developmental outcomes among adolescents without disabilities (Hui et al., 2018; Li et al., 2018; Xin et al., 2019; Chen et al., 2022). However, previous studies have not examined the influence of self-esteem on different sources of perceived social support among adolescents with visual impairment.

Third, the relationship might be reciprocal. However, longitudinal studies examining the possible reciprocal relationship between sighted adolescents’ perceived social support and self-esteem yielded divergent findings. Asendorpf and van Aken (2003) found that support from parents and peers at age 12 predicted global self-worth at age 17, but not vice versa; in contrast, Marshall et al. (2014) reported that self-esteem reliably predicted perceived social support across time in high school students, but not vice versa. As such, the relationships between self-esteem and social support among adolescents must be further examined through longitudinal studies, particularly among adolescents with visual impairment.

### Social integration and perceived social support

According to Wong and Solomon’s (2002) conceptual model of community integration for people with psychiatric disabilities, social integration includes two dimensions—willingness to participate in social interactions and perceived social acceptance from the social network. Social integration herein is conceptualized as visually impaired students’ willingness to be take part in social interactions and their perceived social acceptance from their social network (Xie et al., 2022). Researchers have been concerned about challenges regarding social interaction and social acceptance among children and adolescents with visual impairment for decades (e.g., Skellenger et al., 1990; Lifshitz et al., 2007; de Verdier, 2016; Eguavoen and Eniola, 2016; Jessup et al., 2017; Veerman et al., 2019). There are a few qualitative studies on experiences of students with visual impairments in secondary mainstream schools (West et al., 2004) or universities (Bodaghi et al., 2017; Manyumwa, 2018) that indicated social support might have impact on their social integration. No quantitative study has been found to consider different sources of social support’s relationship with social integration in adolescents with visual impairment.

Social integration (representing the quantity/structure of social relations) and social support (involving their quality/function) are closely associated (Barger, 2013). A series of empirical studies on students without disabilities supports the impact of social support on social integration (e.g., Hoffman et al., 2015; Brunsting et al., 2021). The reverse relationship, from social integration to perceived social support, is also supported. Some scholars believe that individuals with multiple social networks (i.e., better social integration) could perceive more social support (Thoits, 2011). This theoretical proposition has been verified by research on foster youths (Zinn et al., 2017). Furthermore, a longitudinal study on students studying abroad revealed a reciprocal relationship between perceived social support and frequency of students’ interaction through social platforms (Billedo et al., 2019).

While existing studies indicate the close relationships between perceived social support and social integration, they have four limitations to be addressed. First, previous research mainly focuses on social support’s impact on social integration; the reverse path, however, has gained little attention. Second, most studies only focus on a single source of social support or treat social support as a single construct; the relationships between different sources of social support and social integration have remained unexamined. Third, most studies adopt a cross-sectional design; however, more longitudinal studies are needed. Fourth, the relationship between perceived social support and social integration in adolescents with visual impairment has been largely overlooked.

### The present study

Overall, the objectives of the present study were to examine: (1) the reciprocal relationships between perceived social support and self-esteem, and (2) the reciprocal relationships between perceived social support and social integration among Chinese adolescents with visual impairments. This study enriches the literature on the relationships among perceived social support, self-esteem, and social integration and provides theoretical foundations for psychosocial adaptation interventions among adolescents with visual impairment. Based on the above theoretical foundations and empirical evidence, the following four research hypotheses were proposed:

*Hypothesis 1*: Four sources of perceived social support (i.e., parental, teacher, classmate, and close-friend support) at Time 1 (T1) would positively predict self-esteem at Time 2 (T2).

*Hypothesis 2*: Self-esteem at T1 would positively predict four sources of perceived social support at T2.

*Hypothesis 3*: Four sources of perceived social support at T1 would positively predict social integration at T2.

*Hypothesis 4*: Social integration at T1 would positively predict four sources of perceived social support at T2.

## Materials and methods

### Participants and procedures

Ethical approval was granted by the Human Research Ethics Committee of the University of Hong Kong before the data collection. The first author sent, *via* snowballing, invitations to principals of ten special schools that have secondary students with visual impairment, and seven principals agreed to participate. Three smaller schools were selected to conduct the pilot study in order to validate the measurements. The present study is the main study, involving the remaining four schools (three for the visually impaired and one general special school accommodating students with visual impairment and also those with other types of disabilities such as hearing impairment and intellectual disability) in three provinces of eastern China. Secondary stage included three types (i.e., junior high, senior high, and vocational high) in Chinese special schools. Junior high graduates are divided into senior high level or vocational high level. The four schools in the present study are boarding schools. But according to the principals, a small number of students’ homes are near the school, and thus did not live on campus.

First, on the commencement day of fall semester, students and their parents were informed about the study purpose and content and those who agreed to participate signed consent forms. Then, the survey was conducted later in the first month of the semester by gathering students with consent forms in meeting rooms in their schools. The first author restated the study purpose and content and told them their participation was voluntary and their responses would be kept confidential. They were also informed that they could withdraw from the investigation at any time. Then, the first author read the questionnaire aloud, item by item, and the participants responded in their preferred writing medium (Braille or print). The questionnaire included a demographic sheet, and measurements of the research’s constructs. Each participant was offered a chocolate bar after the survey as a small incentive for participation. One year later, the same students were invited to attend the second wave of data collection.

In the first data collection wave, 334 students attended the survey, yielding 311 valid responses. One year later, 177 students participated in the second-wave data collection, 170 of whom were matched with first-wave participants. Demographic information concerning participants’ age and distribution on gender, school stage, onset, and severity of visual impairment is presented in Table 1. The participants’ age fit with Sawyer et al.’ (2018) definition of adolescents as having an age range of 10–24 years. All participants had a disability certificate issued by the China Disabled Persons’ Federation. Blindness and low vision were defined according to the classification and grading system of disability of the China Disabled Persons’ Federation (2011): blindness refers to a best corrected visual acuity of less than 0.05 or a visual field of less than 10 degrees, and low vision represents a best corrected visual acuity of 0.05–0.3. None of the participants had any additional disabilities.

**Table 1:** Demographic information.

Demographic variable		Time 1	Time 2
Age range (*M*, SD)		11 ~ 24(16.15,2.34)	13 ~ 24(17.06,2.32)
Gender	Female	123	71
	Male	188	99
School level	Junior high	162	72
	Senior high	44	36
	Vocational high	105	62
Onset of visual impairment	Congenital	179	98
	Acquired	123	68
	Missing	9	4
Severity of visual impairment	Blind	152	86
	Low vision	154	82
	Missing	5	2
Total number		311	170

Chi-square and *t*-test results showed no significant differences between participants at T1 and T2 in terms of gender, age, school type, time of occurrence of visual impairment, or severity of visual impairment. In addition, MANOVA results showed no significant differences in perceived social support, self-esteem, and social integration between participants at T1 and T2.

### Measures

#### Child and adolescent social support scale

Perceived social support was measured by the Child and Adolescent Social Support Scale (CASSS) developed by Malecki et al. (2000). The CASSS is comprised five 12-item subscales (i.e., parental, teacher, classmate, close friends, and school support), measuring perceived social support of children and adolescents from Grades 3 to 12, with good reliability and validity (Martínez et al., 2011). Based on this study’s purpose, the researchers selected four subscales: parental support (“My parents understand me”), teacher support (“My teachers explain things that I do not understand”), classmate support (“My classmates give me ideas when I do not know that to do”), and close-friend support (“My close friend understands my feelings”). After obtaining consent from Malecki, who developed the CASSS, we translated and adapted it based on the Chinese context. The revised CASSS has four eight-item subscales. Participants rate each item on a six-point Likert scale (1 = never and 6 = always) to indicate the frequency of their perceived support. The internal consistency of each dimension of perceived social support was 0.88 (Parent), 0.87 (Teacher), 0.87 (Classmate), and 0.91 (Close friend) at T1, and 0.88 (Parent), 0.88 (Teacher), 0.86 (Classmate), and 0.90 (Close friend) at T2.

#### Rosenberg self-esteem questionnaire

The Rosenberg self-esteem questionnaire (RSE; Rosenberg, 1965) was adopted to measure self-esteem. The original RSE is a ten-item scale. Kapinga and Aloni (2021) used RSE to assess self-esteem of students with visual impairment in Tanzania, without reporting its psychometric properties. Dodds et al. (1991) validated it among visually impaired adults, resulting in a nine-item version. In the present study, item 9 (“I wish I could have more respect for myself”) was deleted because its factor loading was only 0.07 when confirmatory factor analysis was conducted. Thus, the eight-item version of the RSE was used in this study. Participants used a six-point Likert scale (1 = strongly disagree and 6 = strongly agree) to indicate the degree to which they agreed with each item. The internal consistency of the RSE was 0.74 at T1 and 0.78 at T2.

#### Interpersonal adaptation scale

Social integration was measured using the interpersonal adaptation scale, a nine-item subscale of the Social Adaptability of Second School Students Scale (Chen and Huang, 2004) consisting of two dimensions: willingness to be engaged in social interactions (five items) and perceived social acceptance (four items). The adolescents responded using a six-point Likert scale (1 = strongly disagree and 6 = strongly agree). Since the factor loading of item 4 (“I feel uncomfortable speaking in public”) was 0.05 at T1 when confirmatory factor analysis was performed, it was deleted. The internal consistency of the scale was 0.83 at both time points.

### Data analysis

Two teachers of students with visual impairment who are proficient in Braille were hired to transcribe responses into Excel file, which was then imported into SPSS. Data were analyzed using SPSS 21.0 and Mplus 8.0. First, the longitudinal measurement invariance of each scale was tested using a series of confirmatory factor analyses. Second, data were described by means, standard deviations, skewness, and kurtosis. A repeated-measure multivariate analysis of variance (MANOVA) was used to examine the main effects and interactive effects of time, gender, school type, onset of visual impairment, and severity of visual impairment on the main research variables. Third, Pearson’s correlation was performed to preliminarily examine the perceived relationships among social support, self-esteem, and social integration. Finally, using Mplus 8.0, cross-lagged panel modeling was carried out to examine the reciprocal relationships between the four sources of perceived social support and self-esteem and between the four sources of perceived social support and social integration. Missing data were estimated using full information maximum likelihood (FIML). The robust maximum likelihood estimator (MLR) was used to estimate the model. Chi-square values, CFI, TLI, RMSEA, and SRMR were adopted to evaluate the goodness-of-fit of the cross-lagged model (Hu and Bentler, 1999).

## Results

### Longitudinal measurement invariance

Before testing the reciprocal relationships among the key research variables, the longitudinal measurement invariance of perceived social support, self-esteem, and social integration should be established. The equivalence of factor structure (i.e., configural invariance model), factor loadings (i.e., the metric invariance model), and item intercepts (i.e., scalar invariance model) for the three key constructs and the model comparisons are shown in Table 1. According to Negru-Subtirica et al.’s (2015) criteria, measurement invariance could be established if at least two of the following conditions are met: a significance level of Δ*χ*^2^ > 0.05, ΔCFI ≤ 0.01, and ΔRMSEA ≤ 0.015. Results (see Table 2) supported the configural, metric, and scalar invariance models for the three variables, suggesting cross-lagged panel modeling analysis could be conducted.

**Table 2:** Longitudinal measurement invariance of perceived social support, self-esteem, and social integration.

Model	Fit index						Model comparison	Fit index			
	*χ* ^2^	*df*	CFI	TLI	RMSEA(90%CI)	SRMR		Δ*χ*^2^	Δ*df*	ΔCFI	ΔRMSEA
Perceive social support											
1. Configural invariance model	1460.77, *p* = 0.00	896	0.92	0.92	0.05[0.04–0.05]	0.05					
2. Metric invariance model	1489.78, p = 0.00	924	0.93	0.92	0.05[0.05–0.06]	0.06	1 vs. 2	29.01(*p* > 0.05)	28	<0.01	<0.015
3. Scalar invariance model	1521.60, *p* = 0.00	952	0.93	0.92	0.05[0.05–0.09]	0.06	2 vs. 3	31.82(p > 0.05)	28	<0.01	<0.015
Self-esteem											
4. Configural invariance model	1.05, *p* = 0.96	5	1.00	1.03	0.00 (0.00–0.00)	0.02					
5. Metric invariance model	3.67, *p* = 0.82	7	1.00	1.02	0.00 (0.00–0.04)	0.04	4 vs. 5	2.62(p > 0.05)	2	<0.01	<0.015
6. Scalar invariance model	8.27, *p* = 0.51	9	1.00	1.00	0.00 (0.00–0.06)	0.03	5 vs. 6	4.60(p > 0.05)	2	<0.01	<0.015
Social integration											
7. Configural invariance model	7.27, *p* = 0.20	5	1.00	0.99	0.04 (0.00–0.09)	0.04					
8. Metric invariance model	8.56, *p* = 0.29	7	1.00	0.99	0.03 (0.00–0.08)	0.05	7 vs. 8	1.29(p > 0.05)	2	<0.01	<0.015
9. Scalar invariance model	9.67, *p* = 0.38	9	1.00	1.00	0.02 (0.00–0.07)	0.05	8 vs. 9	1.11(p > 0.05)	2	<0.01	<0.015

### Descriptive statistics

Data were preliminarily analyzed regarding descriptive statistics, zero-order correlations, and repeated-measures MANOVA. First, the research variables’ means and standard deviations are shown in Table 3. The absolute values of skewness and kurtosis of all variables were lower than 1, indicating that they were normally distributed.

**Table 3:** Descriptive statistics and zero-order correlations among perceived social support, self-esteem, and social integration.

	1	2	3	4	5	6	7	8	9	10	11	12
1. T1 parental support	1.00											
2. T1 teacher support	0.46^***^	1.00										
3. T1 classmate support	0.51^***^	0.58^***^	1.00									
4. T1 close-friend support	0.45^***^	0.50^***^	0.62^***^	1.00								
5. T1 self-esteem	0.23^***^	0.22^***^	0.23^***^	0.21^***^	1.00							
6. T1 social integration	0.43^***^	0.35^***^	0.51^***^	0.41^***^	0.31^***^	1.00						
7. T2 parental support	0.61^***^	0.24^**^	0.35^***^	0.31^***^	0.34^***^	0.30^***^	1.00					
8. T2 teacher support	0.26^***^	0.60^***^	0.44^***^	0.37^***^	0.31^***^	0.26^**^	0.36^***^	1.00				
9. T2 classmate support	0.32^***^	0.36^***^	0.53^***^	0.41^***^	0.29^***^	0.35^***^	0.50^***^	0.57^***^	1.00			
10. T2 close-friend support	0.26^***^	0.32^***^	0.34^***^	0.43^***^	0.35^***^	0.38^***^	0.34^***^	0.40^***^	0.53^***^	1.00		
11. T2 self-esteem	0.37^***^	0.20^**^	0.27^***^	0.09	0.48^***^	0.35^***^	0.45^***^	0.30^***^	0.38^***^	0.23^**^	1.00	
12. T2 social integration	0.32^***^	0.28^***^	0.30^***^	0.27^***^	0.25^**^	0.50^***^	0.38^***^	0.34^***^	0.52^***^	0.48^***^	0.44^***^	1.00
*M*	3.72	3.94	3.57	3.91	4.06	3.91	3.66	3.79	3.70	4.08	4.32	4.00
SD	1.03	1.00	0.99	1.15	0.94	1.02	0.96	0.93	0.87	1.04	0.92	0.91

^**^p < 0. 01, and ^***^p < 0. 001.

Second, zero-order correlations of the variables are presented in Table 2. The correlation coefficients of the same variables were moderate and significant at both time points (four dimensions of social support: *r* = 0.43 ~ 0.61, *p* < 0. 001; self-esteem: *r* = 0.48, *p* < 0.001; social integration: *r* = 0.50, *p* < 0.001), indicating moderate stability of perceived social support, self-esteem, and social integration among adolescents with visual impairment. Except for the non-significant relationship between close-friend support at T1 and self-esteem at T2, all correlation coefficients among the four dimensions of perceived social support, self-esteem, and social integration were positively significant at both time points.

Third, the results of repeated-measures MANOVA showed no statistical significant time differences (Wilks’Λ = 0.99, *F*[1, 46] = 0.72, *p* > 0.05, *η*^2^ = 0.02), gender differences (Wilks’Λ = 0.93, *F*[1, 46] = 3.60, *p* > 0.05, *η*^2^ = 0.07), onset of visual impairment differences (Wilks’Λ = 1.00, *F*[1, 46] = 0.17, *p* > 0.05, *η*^2^ = 0.00), and severity of visual impairment differences (Wilks’Λ = 1.00, *F*[1, 46] = 0.06, *p* > 0.05, *η*^2^ = 0.00). Therefore, no demographic factors were controlled in subsequent cross-lagged panel analysis.

### Reciprocal relationships among perceived social support, self-esteem, and social integration

**Figure 1:**
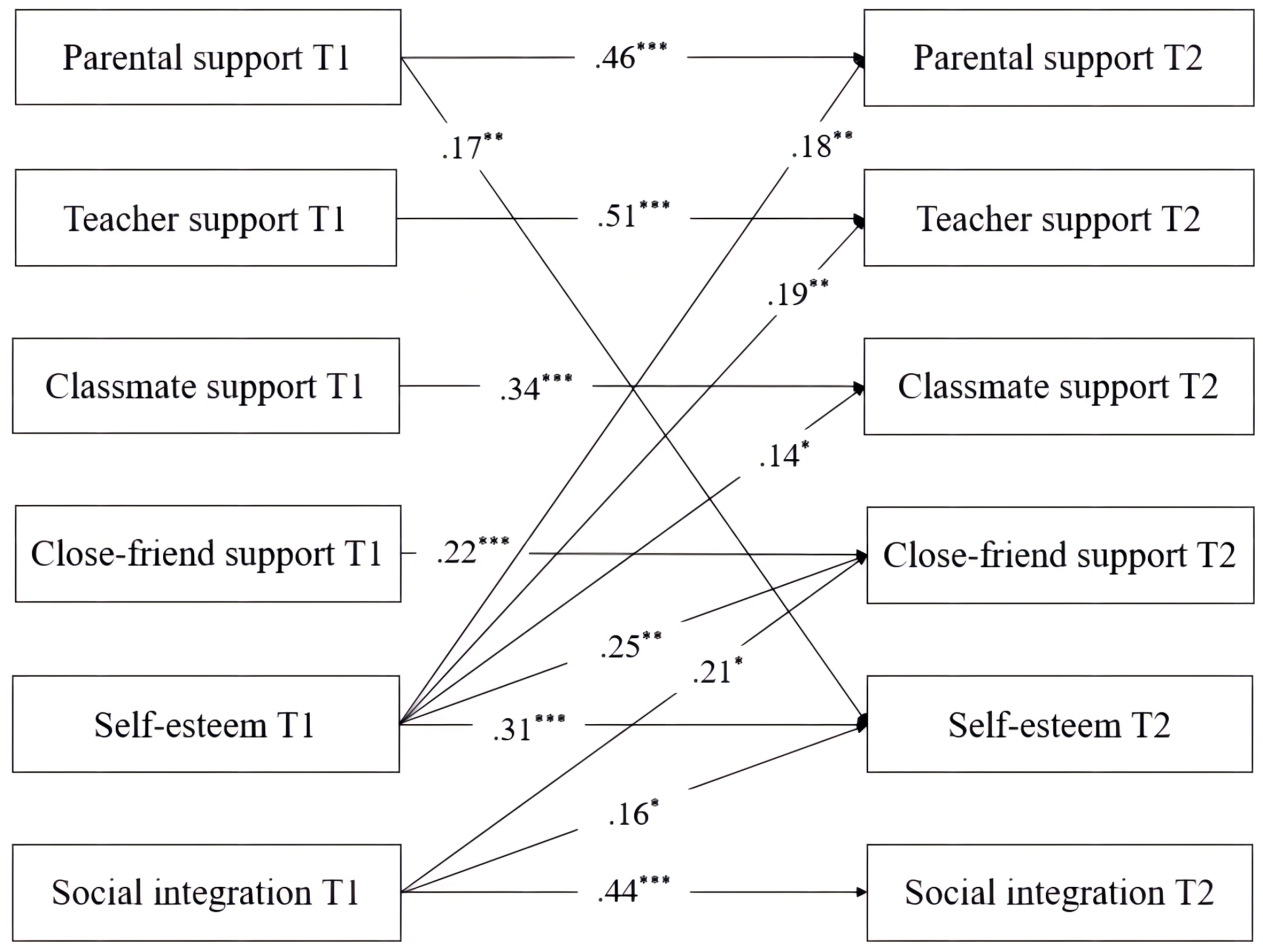
The cross-lagged model of perceived social support, self-esteem, and social integration. ^*^p < 0.05, ^**^p < 0.01, ^***^p < 0.001.

A cross-lagged panel model was constructed to examine the reciprocal relationships between perceived social support and self-esteem and between perceived social support and social integration (see Figure 1). Although the reciprocal relationship between self-esteem and social integration was not a research objective in this study, Xie et al.’s (2022) findings suggest that setting up a cross-lagged path between the two variables would help obtain better model fit and more rigorous results among perceived social support, self-esteem, and social integration. The results suggested that the model fit indices were good: *χ*^2^ = 25.54, *df* = 13, *p* < 0.05, RMSEA = 0.06, SRMR = 0.03, CFI = 0.98, TLI = 0.91. As presented in Figure 1, among the four sources of social support at T1, only parental support significantly and positively predicted T2 self-esteem (*β* = 0.17, *p* = 0.004), partially supporting Hypothesis 1; T1 self-esteem significantly and positively predicted all four sources of social support at T2 (*β*_parent_ = 0.18, *p* = 0.002; *β*_teacher_ = 0.19, *p* = 0.005; *β*_classmate_ = 0.14, *p* = 0.045; *β*_close-friend_ = 0.25, *p* = 0.002), fully supporting Hypothesis 2; no sources of social support at T1 significantly predicted T2 social integration, rejecting Hypothesis 3; T1 social integration only significantly and positively predicted T2 close-friend support (*β* = 0.21, *p* = 0.018), partially supporting Hypothesis 4.

## Discussion

Adopting a two-wave longitudinal design, this study examined the reciprocal relationships between perceived social support and self-esteem and between perceived social support and social integration. The results showed positive reciprocal relationships between parental support and self-esteem. Self-esteem also positively predicted teacher support, classmate support, and close-friend support; however, these three sources of perceived support did not predict self-esteem. Social integration positively predicted close-friend support; however, no sources of perceived social support predicted social integration.

### Relationships between perceived social support and self-esteem

This study partially supported Hypothesis 1 that among the four sources of social support (parental, teacher, classmate, and close-friend support), only parental support significantly and positively predicted self-esteem. The findings support the sociometer theory (Leary, 2005) concerning the view that social support is an important source of self-esteem. The findings confirmed Asendorpf and van Aken’s (2003) cross-lag study results that social relationship quality predicted self-esteem among adolescents, yet diverged from Marshall et al.’s (2014) that adolescents’ social support did not predict self-esteem across time. As Marshall et al. (2014) have discussed, such discrepancies could be attributed to methodological differences in terms of construct operationalizations and data analysis. Another possible explanation is that Marshall et al.’s study examined the overall effect of social support quality and network size, however, the present study examined unique effect of various sources of social support. Therefore, we should be cautious about comparing these results. Above all, no firm conclusions can be drawn at present with respect to the longitudinal effect of perceived social support on self-esteem. Ongoing investigations are necessary to test the results revealed in this study.

With regard to the unique effects of various sources of social support, self-esteem is only found to be the consequence of parental support. This is in line with studies on adolescents without disabilities in China (Liu and Zou, 2007) and the United States (Rueger et al., 2010) which both found that parental support contributed the most to adolescents’ self-esteem from various sources of social support. At the same time, the results aligned with previous findings that teacher and peer support did not predict adolescents’ self-esteem (Rueger et al., 2010). However, the present findings were not consistent with more earlier research on adolescents with visual impairment (Huurre et al., 1999; Kef and Deković, 2004), where peer support was found to have a greater effect than parental support in predicting self-esteem. This inconsistency may result from the different educational placements of adolescents with visual impairments. In Huurre et al.’s (1999) and Kef and Deković’s (2004) studies, adolescents with visual impairments were mostly placed in regular education schools and may have been in a weak position when interacting with their sighted peers; such peer relationships could affect their development of self-concept and thus their levels of self-esteem. In the present study, however, the participants were from special education schools and their peer relationships resembled those among non-disabled adolescents.

In addition, this study supported Hypothesis 2 that adolescents with visual impairment’s self-esteem positively predicted the four sources of perceived social support. This is consistent with previous findings that self-esteem significantly contributes to perceived social support among children and adolescents without disabilities (Marshall et al., 2014; Fu et al., 2017; Xin et al., 2019; Chen et al., 2022). Self-esteem’s significant prediction of perceived social support can be explained by the plasticity hypothesis (Brockner, 1988), which claims that self-esteem influences individuals’ perceptions of others’ evaluation of self, and thus individuals’ social relationships. Holding more positive self-beliefs may boost adolescents’ confidence in relationship management, and thus they become more inclined to develop close relationships, which, in turn, improves their perceived social support (Marshall et al., 2014).

Although neither teacher nor the two types of peer support significantly predicted self-esteem, parental support did. In turn, self-esteem significantly positively predicted four sources of perceived social support, adding new evidence concerning the direction of the relationships between self-esteem and perceived social support. Overall, this study challenges the view that the relationship between self-esteem and perceived social support is unidirectional (Asendorpf and van Aken, 2003; Marshall et al., 2014), supports the reciprocal relationship model between self-esteem and perceived social support among first-year college students (Lee et al., 2014), and further reveals the differences in such relationships among different sources of perceived social support. Future inquiries on adolescents’ social support are encouraged to examine the unique contribution of each source.

### Relationships between perceived social support and social integration

The results showed that four sources of perceived social support did not predict social integration, while social integration positively predicted close-friend support. The findings did not support Hypothesis 3, but partially supported Hypothesis 4, implying that perceived social support could be an outcome rather than an antecedent of social integration among adolescents with visual impairment.

It is concerning that none of the four sources of perceived social support significantly predicted social integration in the present sample, although plenty of previous studies have endorsed perceived social support’s significant role in adolescents’ social integration (e.g., Asendorpf and van Aken, 2003; Brunsting et al., 2021). One possible explanation is that existing studies did not involve adolescents with visual impairments and were conducted in Western countries. This study, however, was conducted in Chinese special education schools. For students with visual impairment attending special schools in China, most of them resided at school, they only went home during public holidays, and they were rarely allowed to go out of school even during weekends. Therefore, their social interactions were highly limited. In such a segregated educational environment, it is difficult for parents, teachers, and peers’ support to influence students’ social integration.

In addition, consistent with previous studies on foster youths (Zinn et al., 2017) and students studying abroad (Billedo et al., 2019), visually impaired adolescents’ social integration positively predicted perceived social support. Specifically, only close-friend support was significantly predicted by social integration. It is possible that adolescents with visual impairment who have higher levels of social integration are more likely to build multiple social networks and develop friendships and then perceive more support from their close friends. As this study is an exploratory study examining the relationship between perceived social support and social integration among adolescents with visual impairments, future studies could further explore this relationship using more rigorous research designs (e.g., collecting data from more time points and controlling relevant variables).

### Limitations and future directions

It should be acknowledged that this study has at least three limitations. First, it had a relatively high attrition rate, which may affect the generalizability of its results. There are three major reasons for the sample attrition: (1) some students left the school at time 2 because of graduation or transferring to another school; (2) some students were on leave; and (3) some students did not write their names correctly and thus their information could not be matched. In order to validate this study with a lower attrition rate, future research could set the tracking time interval within an academic year or exclude participants in their senior year. Second, the study’s participants were from special education schools, and it needs to be verified if the findings are applicable to adolescents with visual impairment in regular education schools. Under different education placement, students with visual impairment would confront different social context, which may change the nature of the relationship between perceived social support and self-esteem and social integration. Third, this study was a two-wave longitudinal study within a 1-year interval. Future studies could consider examining the relationships among the three key variables using more time points. Thereby, researchers could investigate the growth curves and growth rates to gain a more comprehensive understanding of the link between various sources of perceived social support and associated variables.

## Conclusion and implications

This study concludes that self-esteem is an antecedent of four sources of perceived social support, with only perceived parental support positively predicting self-esteem. Social integration is an antecedent of perceived close-friend support, while no perceived social support predicted social integration. These findings enrich our understanding of how different sources of perceived social support relate to adolescents’ self-esteem and social integration.

The findings of the present study have practical implications. The study emphasizes that parental support is of great importance in promoting self-esteem for adolescents with visual impairment in Chinese special schools, and that self-esteem plays a vital role in their perception of social support. Cultivating high self-esteem merits special concern in designing educational programs and counseling services for children and adolescents with visual impairment. Thus, future studies are urged to develop intervention programs concerning improving self-esteem among adolescents with visual impairments through enhancing their social support. Specifically, the importance of parental support on visually impaired adolescents’ self-esteem should be paid more attention. Special education schools could strengthen their collaborations with adolescents’ parents by initiating home-school activities to enhance the communication between visually impaired adolescents and their parents.

Parents also need to be made more aware of the need to support their children with visual impairment. On the one hand, parents could use various methods (e.g., chatting on social media and visiting schools) to improve the frequency of their interactions with their children and know their needs and difficulties, which is conducive to providing necessary support in time. On the other hand, parents are urged to actively learn effective communication skills through self-study or professional training. As parental support is an antecedent of self-esteem of adolescents with visual impairments, parents should take actions since their child is little instead of waiting until adolescence. Parents could get guidance and support through informal and formal approaches. Informal support could be approached from online and offline activities held by parents associations for the visually impaired. In terms of formal support, the Law of the People’s Republic of China on Family Education Promotion (2021) was enacted which requires the government and society to provide guidance, support, and services for family education. Multiple organizations such as Communist Youth League, Disabled Person’s Federations, and China Working Committee for the Care of the Next Generation carry out family education work and provide social support for family education. These organizations should put more effort and concern into empowering parents of children and adolescents with visual impairment.

In addition, the findings indicated that facilitating social integration could help adolescents with visual impairment perceive more close-friend support, benefiting their friendship development. Perceived social support from parents, teachers, classmates, and close friend all failed to contribute to the social integration of adolescents with visual impairment in special schools, indicating that these social relations did not perform their appropriate function. Interventions could be designed to improve the quality of their social relations. Communities could create a more inclusive living environment for people with visual impairments, by improving barrier-free facilities and carrying out community integration activities. Such initiatives could enhance the accessibility of community facilities to individuals with visual impairment and increase opportunities for them to socialize with people who are sighted. Special education schools could collaborate with local communities to organize activities to enhance the interactions between visually impaired adolescents and their sighted peers. Parents could encourage their children to participate in inclusive activities.

## Data availability statement

The raw data supporting the conclusions of this article will be made available by the authors, without undue reservation.

## Ethics statement

The studies involving human participants were reviewed and approved by the Human Research Ethics Committee (HREC) of the University of Hong Kong. Written informed consent to participate in this study was provided by the participants’ legal guardian/next of kin.

## Author contributions

WY contributed to the conceptualization, research design, project administration, data collection, data analysis, and write-up. ZX and PD contributed to the write-up. YY contributed to the revision of the manuscript. All authors contributed to the article and approved the submitted version.

## Funding

This research is partly supported by a grant from the National Natural Science Foundation of China (Grant No. 62107020) and Ministry of Education of the People’s Republic of China (Grant No. 21YJA880078).

## Conflict of interest

The authors declare that the research was conducted in the absence of any commercial or financial relationships that could be construed as a potential conflict of interest.

## Publisher’s note

All claims expressed in this article are solely those of the authors and do not necessarily represent those of their affiliated organizations, or those of the publisher, the editors and the reviewers. Any product that may be evaluated in this article, or claim that may be made by its manufacturer, is not guaranteed or endorsed by the publisher.
